# Risperidone-Induced Amenorrhea in Floridly Psychotic Female

**DOI:** 10.7759/cureus.1683

**Published:** 2017-09-13

**Authors:** Shanila Shagufta, Faiza Farooq, Ali M Khan, Kamil Dar, Abdul Mohit

**Affiliations:** 1 New York Medical College, Kings County Hospital Center; 2 Psychiatry, Kings County Hospital Center; 3 Psychiatry, Services Hospital, Services Institute of Medical Sciences, Lahore, Pakistan; 4 Behavioral Health, Kings County Hospital Center

**Keywords:** risperidone, amenorrhea, psychosis

## Abstract

Despite the high prevalence of hyperprolactinemia in patients receiving antipsychotic medications, its side effects are often neglected. In patients receiving risperidone, the incidence of menstrual abnormalities is relatively small. Our patient was a 44-year-old, Haitian female whose total course of hospitalization was nine months, during most of which she remained floridly psychotic with low cognitive function with waxing and waning symptoms. She developed hyperprolactinemia and amenorrhea on risperidone. She was treated and discharged to the state mental hospital. Menstrual abnormalities cause psychological distress in women. In women, hyperprolactinemia can cause sexual and reproductive dysfunction. Chronic hyperprolactinemia can predispose to osteoporosis and cardiovascular disease. Clinicians should be vigilant about the consequences when prescribing medications for women, particularly those suffering from a psychotic disorder.

## Introduction

The prevalence of risperidone-induced amenorrhea is only 1%-10% [1]. The sexual and reproductive side effects of atypical antipsychotics contribute to 50% of the noncompliance reported for treated patients. Hyperprolactinemia is defined as plasma levels of prolactin >20ng/ml in males and 25ng/ml in females. In women, this leads to menstrual abnormalities, galactorrhea, infertility, and sexual dysfunction. In men, it can cause erectile dysfunction, decreased libido, and gynecomastia [2-3].

Many pathophysiological conditions can lead to hyperprolactinemia, like pregnancy, medication side effects, or hypothyroidism. The prolactin is secreted by the lactotroph cells in the anterior pituitary. Its synthesis and production are controlled by neurotransmitters, steroids, and peptides. Dopamine binds to the lactotroph cells and inhibits prolactin secretion. All antipsychotics block D2 receptors and induce hyperprolactinemia [2-4].

Typical antipsychotics non-electively bind to the dopamine receptors in all areas of the brain. They reduce the positive symptoms (hallucinations, delusions, and bizarre behavior) by antagonizing dopamine receptors in the limbic system and hence raise prolactin levels. The atypical antipsychotics (clozapine, risperidone, quetiapine, olanzapine) have a higher affinity for serotonin than dopamine receptors. These agents are called serotonin-dopamine antagonists (SDAs). However, risperidone is characterized by a stronger affinity for D2 receptors and hence increases prolactin levels.

Some of the adverse effects of hyperprolactinemia are caused by its direct effect on the tissue. The hypogonadism caused by prolactin disrupts the hypothalamic-pituitary axis. The hypothalamus releases pulsatile gonadotropin releasing hormone (GnRH); this results in normal secretion of the luteinizing hormone (LH) and follicular stimulating hormone (FSH)  by the pituitary gland. The pulsatile release causes follicular growth and secretion of estrogen.The peak estrogen level initiates a LH surge for ovulation and the formation of corpus luteum. If no pregnancy occurs, the prostaglandins F2 alpha from the uterus causes luteolysis and thus starts another menstrual cycle [2-5].

The lack of pulsatile release of GnRH leads to an anovulatory stage with periods being irregular. It can progressively lead to impaired secretion of LH and FSH and can prevent a normal ovarian response. Eventually, it can cause a hypoestrogenism amenorrheic cycle leading to infertility [6].

## Case presentation

The patient was a 44-year-old, Haitian female with a past psychiatric history of paranoid schizophrenia. The patient lived by herself. She was initially brought to the hospital because she refused to come out of her room to eat. She only drank water for a month. She was scared to go out and buy food as she thought "people were banging their heads on the wall". At the time of her admission, she presented unkempt and withdrawn with decreased appetite, anhedonia, speech latency, and poverty of thought and content. She was noncompliant to medications and was unable to take care of herself. Due to starvation in the context of psychosis (paranoia, fear of going out), she developed lactic acidosis (pH=4.3) and hypokalemia (2.5 meq/l) and was initially treated in the medical unit before being transferred to the psychiatric unit. The patient had prior hospitalizations for similar presentations and was treated with oral risperidone 3 mg twice daily, oral escitalopram once daily, and oral valproate 1,000 mg once daily. She had no history of substance abuse. The total course of her hospitalization was nine months. She became suspicious of her medications and reported auditory hallucinations, so she was started on oral risperidone 3 mg once daily. The patient's paranoia, as she stated the "staff is putting poisonous powder on my skin", improved, and she was seen eating and sitting with others in the TV room. Although, the patient was responding well to risperidone, given her history of non-compliance (which led to her hypokalemia and lactic acidosis) and ambivalence to taking oral medications, the team decided to put her on long-acting Risperdal Consta 50 mg/2 weeks. The patient's symptoms improved: she became more interactive, attended groups, and started eating better, but she was still paranoid and responding to internal stimuli, occasionally. She remained an inpatient for four months and received six doses of Risperdal Consta 50 mg intramuscular (IM) injections. She developed hyperprolactinemia (prolactin level = 53.32, N = 20ng/ml) though she denied having any spontaneous milky nipple discharge. The patient was then asked about menstrual irregularities, and she reported having no periods for three months. Although her psychosis improved with risperidone, she remained guarded and paranoid throughout her hospitalization. Since she had hyperprolactinemia and amenorrhea, the team decided to discontinue the risperidone, and the patient was started on monthly Abilify Maintenna 400 mg intramuscular injections. She tolerated it well. She received two consecutive doses of Abilify Maintena. Her amenorrhea resolved, and she was discharged to the state hospital.

## Discussion

Secondary amenorrhea due to hyperprolactinemia occurs in 30% of pre-menopausal women on risperidone. Although the occurrence of risperidone-induced hyperprolactinemia and reproductive dysfunction is relatively common, it is particularly challenging to evaluate it in grossly disorganized and psychotic patients. This case highlights risperidone-induced hyperprolactinemia that resulted in amenorrhea. The patient's prolactin level was elevated to 53.32 ng/ml after she was placed on Risperdal Consta for three months; simultaneously, she developed amenorrhea which was reported by the patient three months later. Utilizing the Naranjo scale (drug adverse reaction rating scale), the risperidone-induced hyperprolactinemia was rated probably [7]. Risperidone is metabolized by the cytochrome p450 2D6 pathway in the liver to its active metabolite 9-hydroxy risperidone. The selective serotonin reuptake inhibitors (SSRIs) can inhibit p450 2D6 and can increase risperidone levels. However, escitalopram is a weak inhibitor and does not have a clinically significant effect on the risperidone level [8]. The hyperprolactinemia due to antipsychotic medications is dose dependent and more commonly seen in women. Although the patient was on risperidone 3 mg orally twice daily, she developed hyperprolactinemia after being on Risperdal Consta 50 mg IM/bi-monthly. It is interesting to note that the degree of prolactin elevation is not a predictor of developing side effects. Although her prolactin level was moderately elevated (prolactin = 53.32ng/ml), she still developed amenorrhea. The development of amenorrhea in premenopausal women can lead to unintended pregnancy, as the patient may surmise they have menopause and do not need contraception. In men, this may present as sexual dysfunction or visual problems. Overall, the prevalence of sexual dysfunction due to antipsychotics is reportedly between 18% to 96%. The symptoms of sexual and reproductive dysfunction are often under reported and unexplored in clinical practice. However, they are a major reason for nonadherence in patients treated with antipsychotic medications [3-9].

## Conclusions

Most patients diagnosed with a psychiatric disorder are placed on lifelong antipsychotic medications. Clinicians should evaluate for symptoms of hyperprolactinemia and its severity and frequency, which can be challenging in patients suffering from a psychotic disorder. This will lead to appropriate management and treatment regimen for patients who are on lifelong therapies. It will also alleviate psychological distress in patients and will improve compliance to medications.
